# Ability of minimally invasive surgery to decrease incisional surgical site infection occurrence in patients with colorectal cancer and other gastroenterological malignancies

**DOI:** 10.3389/fsurg.2023.1150460

**Published:** 2023-04-12

**Authors:** Takehito Yamamoto, Mami Yoshitomi, Yoshiki Oshimo, Yuta Nishikawa, Koji Hisano, Kenzo Nakano, Takayuki Kawai, Yoshihisa Okuchi, Kohta Iguchi, Eiji Tanaka, Meiki Fukuda, Kojiro Taura, Hiroaki Terajima

**Affiliations:** ^1^ Department of Gastroenterological Surgery and Oncology, Medical Research Institute KITANO HOSPITAL, Osaka, Japan; ^2^ Department of Surgery, Hyogo Prefectural Amagasaki General Medical Center, Amagasaki, Japan

**Keywords:** colorectal cancer, laparoscopic surgery, minimally invasive surgery, surgical site infection, hyperglycemia, SSI

## Abstract

**Background:**

Surgical site infection (SSI) is one of the most important complications of surgery for gastroenterological malignancies because it leads to a prolonged postoperative hospital stay and increased inpatient costs. Furthermore, SSI can delay the initiation of postoperative treatments, including adjuvant chemotherapy, negatively affecting patient prognosis. Identifying the risk factors for SSI is important to improving intra- and postoperative wound management for at-risk patients.

**Methods:**

Patients with gastroenterological malignancies who underwent surgery at our institution were retrospectively reviewed and categorized according to the presence or absence of incisional SSI. Clinicopathological characteristics such as age, sex, body mass index, malignancy location, postoperative blood examination results, operation time, and blood loss volume were compared between groups. The same analysis was repeated of only patients with colorectal malignancies.

**Results:**

A total of 528 patients (330 men, 198 women; mean age, 68 ± 11 years at surgery) were enrolled. The number of patients with diseases of the esophagus, stomach, small intestine, colon and rectum, liver, gallbladder, and pancreas were 25, 150, seven, 255, 51, five, and 35, respectively. Open surgery was performed in 303 patients vs. laparoscopic surgery in 225 patients. An incisional SSI occurred in 46 patients (8.7%). Multivariate logistic regression analysis showed that postoperative hyperglycemia (serum glucose level ≥140 mg/dl within 24 h after surgery), colorectal malignancy, and open surgery were independent risk factors for incisional SSI. In a subgroup analysis of patients with colorectal malignancy, incisional SSI occurred in 27 (11%) patients. Open surgery was significantly correlated with the occurrence of incisional SSI (*P* = 0.024).

**Conclusions:**

Postoperative hyperglycemia and open surgery were significant risk factors for SSI in patients with gastroenterological malignancies. Minimally invasive surgery could reduce the occurrence of incisional SSI.

## 1. Introduction

Surgical site infection (SSI) is one of the largest contributors to overall inpatient costs (1–5). Furthermore, especially in patients with gastroenterological malignancies, SSI can lead to delayed initiation of postoperative treatment and negatively affect patient prognosis. Determining the risk factors for SSI could potentially improve intra- and postoperative wound management in at-risk patients.

Many studies have reported different pre-, intra-, and postoperative risk factors for SSI in abdominal surgery (6–8), such as diabetes, perioperative transfusion, cirrhosis, and bowel anastomosis. Numerous studies have reported that patients with diabetes are prone to postoperative infectious diseases, including SSI (9–11). Martin et al. reported an association between diabetes and the risk of SSI in a large systematic review and meta-analysis (9). In contrast, Ata et al. revealed that a high serum glucose level was the only significant predictor of SSI in colorectal surgery patients, which means that postoperative hyperglycemia, rather than diabetes mellitus itself, affects the occurrence of SSI in known as well as unknown or non-diabetic patients (12).

Minimally invasive surgery, including laparoscopic or robotic surgery, recently became the standard treatment for many types of cancer. Minimally invasive surgery can reduce the occurrence of SSI. Kagawa et al. reported an association between increased use of the laparoscopic approach and decreased SSI rates during a 13-year study period (13).

The guidelines of the Centers for Disease Control and Prevention (CDC) divide SSI into three types: incisional, deep, and organ/space (14). Although previous studies discussed all SSI types, we believe that the risk factors and preventive measures for each of the three types differ (15). Therefore, in the present study, we focused on incisional SSI and its risk factors among patients who underwent gastroenterological surgery for malignancies.

## 2. Materials and methods

### 2.1. Study participants

Patients with gastroenterological malignancies who underwent surgery in our hospital between 2011 and 2013 were reviewed. The gastroenterological malignancies included malignant diseases (preoperative diagnoses of cancer, neuroendocrine tumors, and gastrointestinal stromal tumors) of the esophagus, stomach, small intestine, colon and rectum, liver, gallbladder, biliary duct, and pancreas. Based on the concept that SSI occurrence was associated with the operative procedure rather than the disease's location, patients with disease of the distal biliary duct and papilla of Vater who received pancreatic resection were classified as “pancreas.” We then divided the study patients into SSI and no-SSI groups according to the occurrence of incisional SSI and compared the clinical characteristics between them. Furthermore, we conducted the same comparison for a subgroup of only patients with colorectal malignancies.

Patients with diabetes mellitus were defined as those who received pharmacological treatment for diabetes before the operation or a higher than normal hemoglobin A1c level. Postoperative hyperglycemia was defined as a serum glucose level of >140 mg/dl within 24 h postoperative. Current smokers were defined as individuals who smoked within 1 month preoperative.

### 2.2. Diagnosis of incisional SSI

Patients with incisional SSI based on the CDC guidelines were included in the SSI group (16). The wound was examined by a doctor and nurse at least once daily until hospital discharge. After discharge, the wound was examined by an outpatient doctor for 30 days postoperative. SSI was diagnosed after discussion with surgeons, nurses, and members of the SSI surveillance team.

### 2.3. Statistical analysis

Continuous variables are presented as mean ± standard deviation or median [range], and categorical variables were presented as numbers and percentages. The *χ*^2^ and Wilcoxon rank sum tests were used to compare groups. Variables potentially associated with SSI in the univariate analysis (values of *P* < 0.1) were included in the multivariate logistic regression model. The effects of the associations are expressed as odds ratios (OR) and 95% confidence intervals (CI). All statistical analyses were conducted by a participating physician (TY) using JMP version 16 (SAS Institute, Cary, NC, United States). All reported *P*-values were two-sided; those <0.05 were considered statistically significant. The study design was approved by the Ethics Review Board of Kitano Hospital (no. 2201013).

## 3. Results

A total of 528 patients were enrolled. Table 1 shows the clinical characteristics of all participants (330 men, 198 women; mean age, 68 ± 11 years at surgery). The mean body mass index (BMI) was 22.6 ± 3.7 kg/m^2^. The number of patients with diseases of the esophagus, stomach, small intestine, colon and rectum, liver, gallbladder, and pancreas were 25, 150, seven, 255, 51, five, and 35, respectively. Open surgery was performed in 303 patients vs. laparoscopic surgery in 225 patients. Intraoperative blood transfusions were performed in 52 patients.

**Table 1:** Clinical characteristics of all patients.

Variable	N = 528
Age (years)	68 ± 11
Male/female	330 (63)/198 (37)
BMI (kg/m^2^)	22.6 ± 3.7
Diabetes mellitus	87 (17)
Current smoker	51 (10)
Albumin (mg/dl)	4.1 ± 0.5
Postoperative serum glucose, mg/dl	143 ± 41
ASA classification	
I	48 (9)
II	401 (76)
III	78 (15)
IV	1 (0.2)
Organ	
Esophagus	25 (5)
Stomach	150 (28)
Intestine	7 (1)
Colon and rectum	255 (48)
Liver	51 (10)
Gallbladder	5 (1)
Pancreas	35 (7)
Emergency surgery	10 (2)
Surgical approach	
Open	303 (57)
Laparoscopic	225 (43)
Operation time (min)	295 [0–1,039]
Blood loss (ml)	163 [0–4,177]
Intraoperative blood transfusion	52 (10)

Data are presented as mean ± standard deviation, median [range], or *n* (%).

ASA, american society of anesthesiologists; BMI, body mass index.

**Figure 1:**
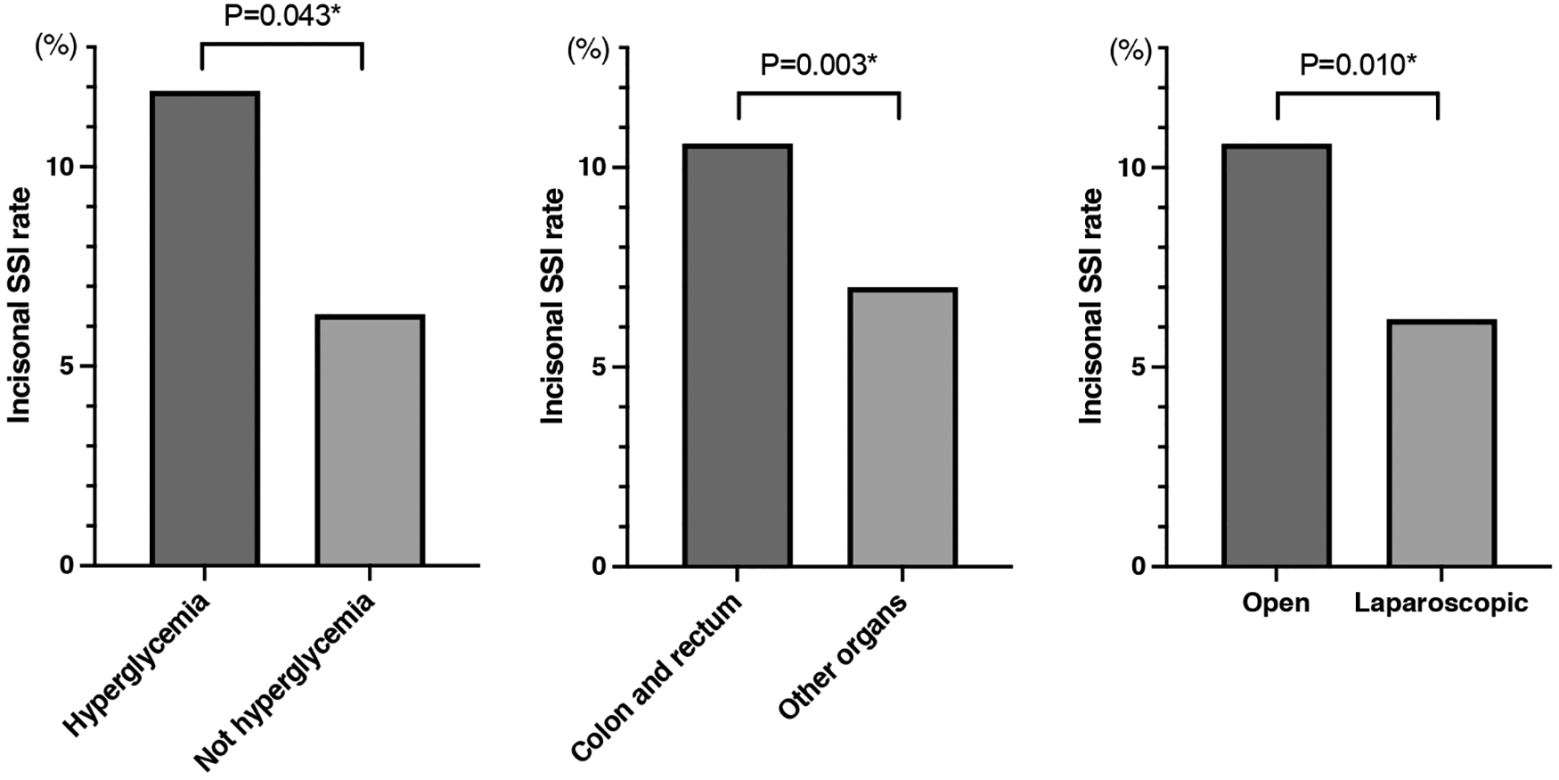
Comparison of the incisional SSI rate by glycemia status, affected organs, and surgical approach. SSI, surgical site infection

An incisional SSI occurred in 46 patients (8.7%). Table 2 compares the SSI and non-SSI groups. A univariate analysis showed that significantly more patients in the SSI group had postoperative hyperglycemia (*P* = 0.024) and that male sex, higher BMI, and open surgery tended to affect the occurrence of incisional SSI (*P* = 0.094, 0.058, and 0.080, respectively). As shown in Table 3, the multivariate logistic regression analysis showed that postoperative hyperglycemia (OR, 1.94; 95% CI, 1.02–3.70; *P* = 0.043), colorectal malignancy (OR, 3.09; 95% CI, 1.49–6.44; *P* = 0.003), and open surgery (OR, 2.73; 95% CI, 1.27–5.89; *P* = 0.010) were independent risk factors for incisional SSI. Based on these three factors, the incisional SSI rates of the two groups are shown in Figure 1.

**Table 2:** Clinical characteristics of the SSI vs. no-SSI groups.

Variable	SSI group	No-SSI group	*P*-value
	*n* = 46	*n* = 482	
Age (years)	68 ± 11	68 ± 11	0.945
Sex			0.094
Male	34 (10)	296 (90)	
Female	12 (6)	186 (94)	
BMI (kg/m^2^)	22.5 ± 3.6	23.8 ± 4.3	0.058
Diabetes mellitus	9 (10)	78 (90)	0.555
Current smoker	5 (10)	46 (90)	0.771
Albumin (mg/dl)	4.0 ± 0.6	4.1 ± 0.5	0.296
Postoperative hyperglycemia	27 (12)	200 (88)	0.024*
ASA classification ≥III	9 (11)	70 (89)	0.360
Organ			0.100
Esophagus	1 (4)	24 (96)	
Stomach	6 (4)	144 (96)	
Intestine	0	7 (100))	
Colon and rectum	27 (11)	228 (89)	
Liver	5 (24)	46 (76)	
Gallbladder	1 (20)	4 (80)	
Pancreas	6 (17)	29 (83)	
Emergency surgery	2 (20)	8 (80)	0.201
Operative procedure			0.080
Open	32 (11)	271 (89)	
Laparoscopic	14 (6)	211 (94)	
Operation time (min)	295 [0–1,039]	288 [107–879]	0.824
Blood loss (ml)	389 [0–1,730]	295 [0–1,039]	0.143
Intraoperative blood transfusion	6 (12)	46 (88)	0.447

ASA, american society of anesthesiologists; BMI, body mass index.

* *P* < 0.05. Data are presented as mean ± standard deviation, mean [range], or *n* (%).

**Table 3:** Multivariate logistic regression analysis of the risk factors for incisional SSI.

Variable	OR	95% CI	*P*–value
Male sex	1.86	0.92–3.76	0.084
BMI > 25 kg/m^2^	1.43	0.72–2.83	0.309
Postoperative hyperglycemia	1.94	1.02–3.70	0.043*
Colon and rectum	3.09	1.49–6.44	0.003*
Open surgery	2.73	1.27–5.89	0.010*

BMI, body mass index; CI, confidence interval; OR, odds ratio; SSI, surgical site infection.

* *P* < 0.05.

In a subgroup analysis of patients with colorectal malignancy, incisional SSI occurred in 27 (11%) patients. Table 4 compares the SSI and no-SSI patients. Open surgery was significantly associated with the occurrence of incisional SSI vs. laparoscopic surgery (*P* = 0.024), while postoperative hyperglycemia tended to affect the occurrence of incisional SSI (*P* = 0.051).

**Table 4:** Clinical characteristics of SSI vs. no-SSI groups among patients who underwent colorectal surgery (*n* = 255).

Variable	SSI group	No-SSI group	*P*-value
	*n* = 27	*n* = 228	
Age (years)	67 ± 12	68 ± 11	0.356
Sex			0.446
Male	17 (12)	126 (88)	
Female	10 (9)	102 (91)	
BMI (kg/m^2^)	22.5 ± 3.5	23.8 ± 4.9	0.225
Diabetes mellitus	6 (17)	26 (83)	0.109
Current smoker	2 (12)	15 (88)	0.870
Albumin (mg/dl)	4.0 ± 0.7	4.1 ± 0.5	0.937
Postoperative hyperglycemia	15 (16)	79 (84)	0.051
ASA classification ≥III	3 (7)	39 (93)	0.427
Emergency surgery	2 (22)	7 (78)	0.248
Operative procedure			0.024*
Open	13 (17)	62 (83)	
Laparoscopic	14 (8)	166 (92)	
Operation time (min)	258 [50–704]	249 [107–715]	0.392
Blood loss (ml)	50 [0–1,100]	148 [0–4,177]	0.300
Intraoperative blood transfusion	2 (3)	6 (97)	0.178

ASA, american society of anesthesiologists; BMI, body mass index.

* *P* < 0.05. Data are presented as mean ± standard deviation, median [range], or *n* (%).

## 4. Discussion

Numerous studies have analyzed the risk factors for SSI in patients with different diseases. Especially in gastroenterology, risk factors and preventive measures for SSI have been widely discussed because of the high rate of SSI.

We found that postoperative hyperglycemia independently affected the occurrence of incisional SSI. Despite ample evidence showing that perioperative hyperglycemia is associated with postoperative infective complications, these studies analyzed all infectious diseases, including SSI, pneumonia, and urinary tract infections (17–19). Previous reports that focused on the association between postoperative hyperglycemia and SSI rate highlighted specific surgical procedures, such as colorectal surgery, cardiothoracic surgery, and sleeve gastrectomy (10, 11, 20). On the other hand, Ata et al. analyzed a total of 2,090 general and vascular surgery patients in whom only the postoperative serum glucose level was associated with SSI (12). Although this result was consistent with the present findings, the study included a diverse array of surgeries for many diseases: clean or dirty, benign or malignant, and gastroenterological or cardiovascular. Our study selected patients with a common circumstance of gastroenterological malignancies, an inclusion criterion that was reasonable for improving postoperative wound management provided by medical personnel in the same department.

In addition, compared to laparoscopic surgery, open surgery was an independent risk factor for incisional SSI, consistent with the results of previous reports (21, 22). Wang et al. reported in a large systematic review and meta-analysis that the laparoscopic approach significantly reduced the SSI rate in gastrointestinal surgeries (21).

In the present study, patients who underwent colorectal surgery were exclusively analyzed as subgroups. Although the subgroup results were slightly different from those of all study patients, open surgery was also the principal risk factor for incisional SSI. Specifically, several studies have reported that use of the laparoscopic vs. open approach in colon cancer patients could reduce the SSI rate (13, 22–25). For example, a large-scale study of 229,726 colorectal cancer patients revealed that the SSI rate was significantly lower in laparoscopic than open surgery, with an OR of 0.43 (26). Similarly, Kulkarni et al. discussed incisional SSI as being separate from other types of SSI and showed that laparoscopic surgery could reduce the rate of all SSI types (25). One could theorize that, especially in open colorectal surgery, a longer incision on the skin will be more prone to intraoperative contamination by fecal ascites than laparoscopic surgery. Therefore, minimally invasive surgery, including laparoscopic surgery, decreases the incisional SSI rate, especially among patients with colorectal cancer.

In this subgroup analysis, postoperative hyperglycemia tended to be associated with incisional SSI. Although several previous studies have analyzed patients who underwent elective colorectal surgery, they have not focused on postoperative serum blood glucose levels, preferring to evaluate the presence or absence of diabetes (27–29). Thus, the results of our subgroup analysis should be of further value for preventing SSI after colorectal surgery.

Unfortunately, in our retrospective study, data on intraoperative body temperature were not available and we were unable to conduct a detailed analysis of the correlation between this factor and SSI occurrence. The guidelines for safe surgery published by the World Health Organization describe that “maintenance of normothermia during surgery” reduces the SSI rate (30). Reports have shown an association between intraoperative hypothermia and SSI incidence (31, 32). Tsuchida et al. reported that severe hypothermia (<35.0°C) and late-nadir hypothermia (<36°C for >2 h after anesthesia induction) were significant risk factors for SSI in prolonged gastroenterological surgery (31). On the other hand, contrasting results were also reported by some studies (33, 34), and the appropriate intraoperative body temperature remains a controversial aspect. Further studies of the impact of intraoperative hypothermia on SSI are warranted.

One measure to reduce SSI is to build a preventive SSI care bundle (i.e., use of systematic approaches). Many reports have indicated the effectiveness of these bundles, some of which include strict glycemic control (15, 35–38). In our hospital, severely diabetic patients were admitted more than 1 week preoperatively and should receive specific management of blood glucose levels by diabetes physicians to reduce potential perioperative complications caused by hyperglycemia. The results reported herein indicate the importance of postoperative management of blood glucose levels for both patients with severe diabetes and for all patients with gastroenterological malignancies.

Our study had several limitations. First, it was conducted at a single center and included a small number of patients. Thus, a large-scale multicenter study is needed to confirm our findings. Second, operative and postoperative management involving sutures or dressings and perioperative antibiotic administration are dependent on different clinicians result in an inconsistent quality of care. Finally, other factors not included in our analysis may have influenced the outcomes.

In conclusion, postoperative hyperglycemia and open surgery are significant risk factors for incisional SSI in patients with gastroenterological malignancies. Meticulous management of postoperative serum glucose levels can positively affect outcomes.

## Data Availability

The raw data supporting the conclusions of this article will be made available by the authors, without undue reservation.
